# Inhibition of Topoisomerase (DNA) I (TOP1): DNA Damage Repair and Anticancer Therapy

**DOI:** 10.3390/biom5031652

**Published:** 2015-07-22

**Authors:** Yang Xu, Chengtao Her

**Affiliations:** School of Molecular Biosciences, College of Veterinary Medicine, Washington State University, Mail Drop 64-7520, Pullman, WA 99164, USA; E-Mail: davidxy22@vetmed.wsu.edu

**Keywords:** topoisomerase (DNA) I (TOP1), anticancer therapy, DNA replication, topoisomerase inhibitor, DNA double-strand break (DSB), DSB repair, homologous recombination (HR), non-homologous end joining, single-strand break (SSB) repair, one-ended DSB

## Abstract

Most chemotherapy regimens contain at least one DNA-damaging agent that preferentially affects the growth of cancer cells. This strategy takes advantage of the differences in cell proliferation between normal and cancer cells. Chemotherapeutic drugs are usually designed to target rapid-dividing cells because sustained proliferation is a common feature of cancer [1,2]. Rapid DNA replication is essential for highly proliferative cells, thus blocking of DNA replication will create numerous mutations and/or chromosome rearrangements—ultimately triggering cell death [3]. Along these lines, DNA topoisomerase inhibitors are of great interest because they help to maintain strand breaks generated by topoisomerases during replication. In this article, we discuss the characteristics of topoisomerase (DNA) I (TOP1) and its inhibitors, as well as the underlying DNA repair pathways and the use of TOP1 inhibitors in cancer therapy.

## 1. Type IB Topoisomerases and Inhibitors

### 1.1. TOP1

**Figure 1:**
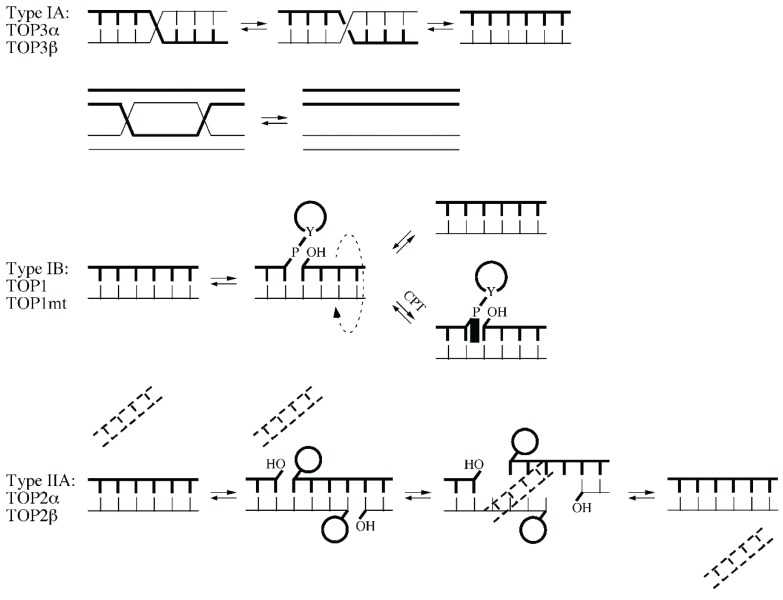
A schematic representation of strand passages catalyzed by three types of topoisomerases (adapted from ref. [8]).

DNA topoisomerases resolve topological constraints that may arise from DNA strand separation and are therefore important for transcription and replication [4]. There are six topoisomerases in humans, classified as Type IA, IB and IIA. Type IA topoisomerases TOP3α and TOP3β cleave one DNA strand to relax only negative supercoiling. In addition, TOP3α forms the BTR complex with BLM and RMI1/2, which plays a role in the dissolution of double-Holliday junctions [5]. Type IIA topoisomerases TOP2α and TOP2β generate double-strand breaks on one DNA molecule to allow the passing of other DNA strands [6]. Topoisomerases are attractive drug targets in cancer therapy. For example, the commonly used anticancer agents doxorubicin and etoposide (VP-16) are TOP2 inhibitors [7]. Type IB topoisomerases include the nuclear TOP1 and mitochondrial TOP1mt [4]. TOP1 initiates the DNA relaxation by nicking one DNA strand. It then forms a TOP1-DNA cleavage complex (TOP1cc) by covalently linked to the 3'-phosphate end via its tyrosine residue Y723 (3'-P-Y). Following the resolution of topological entanglements and the removal of TOP1, the 5'-hydroxyl end is realigned with the 3'-end for religation. Each nicking-closing cycle enables the relaxation of one DNA supercoiling (Figure 1).

TOP1 is essential for embryonic development in mammals [9]. Although TOP1 plays an important role in the deconvolution of supercoils arising amid DNA replication, the precise steps involved with the recruitment of TOP1 to topological constraints remains to be revealed. It appears that in yeast TOP1 travels at a distance of 600 bp ahead of the replication fork [10] and remains associated with the GINS-MCM complex [11]. However, the yeast TOP1 is distinct from its human counterpart in that it has little effect on fork progression or the firing of replication origin [12]. In humans, TOP1 binds to the regions of the pre-replicative complex in cells during the M, early G1, and G1/S phases of the cell cycle to control the firing of replication origins [12]. This difference may explain why yeast cells are viable in the absence of TOP1. In addition, TOP1 also has functions in transcription that are independent of its role in resolving DNA topological entanglements. First, TOP1 is known to repress transcription by binding to TFIID [13]. Second, inhibition of TOP1 can cause the induction of c-Jun in leukemia cells, suggesting its additional role in the control of transcription [14]. Furthermore, TOP1 interacts with the splicing factor ASF/SF2 by which it promotes the maturation of RNA—through suppressing the formation of R-loops (RNA-DNA hybrids)—and prevents collision between transcription bubble and replication fork [15,16]. It appears that the levels of TOP1 have to be dynamically regulated. In B cells, TOP1 is reduced by activation-induced cytidine deaminase (AID) to facilitate class-switch recombination (CSR) and somatic hypermutation (SHM) [17,18]. Although TOP1mt is important for mitochondrial integrity and metabolism, mice lacking mitochondrial TOP1mt are viable and fertile but they are associated with increased negative supercoiling of mtDNA [19,20].

### 1.2. TOP1 Inhibitors

Stabilization of TOP1cc by topoisomerase poison is detrimental to cells due to the disruption of DNA uncoiling, increased strand breaks, and unstable RNA transcripts as well as incomplete DNA replication [21]. The TOP1 inhibitor camptothecin (CPT), first isolated from the Chinese tree *Camptotheca acuminate*, was clinically used for cancer treatment long before it was identified as a TOP1 inhibitor [22]. Due to side effects, CPT is no longer used clinically and it has been replaced by more effective and safer TOP1 inhibitors [23]. Currently, CPT derivatives topotecan (trade name: Hycamtin) and irinotecan (CPT-11, trade name: Camptosar) are routinely used to treat colorectal, ovarian and lung cancers, while a few other TOP1 inhibitors are being tested in clinical trials.

CPT is a 5-ring alkaloid that is active in its closed E-ring (lactone) form but it is inactive with an open E-ring (carboxylate) at physiological and alkaline pH [24]. Therefore, CPT is not effective for inhibiting TOP1mt due to a higher pH mitochondrial environment. The inactive form of CPT tends to bind to serum albumin, which might be a reason for its side effects. CPT is highly specific for TOP1 and the binding is of relatively low affinity and can be reversed after drug removal. These features make the action of CPT controllable [24], and in fact CPT is widely used in studies of replication-associated DNA damage response. There are a few CPT derivatives and non-CPT TOP1 inhibitors [4,8,24]. For example, CPT derivatives Diflomotecan and S39625 were designed to stabilize the E-ring. Irinotecan has the bis-piperidine side chain to increase its water solubility, but it also contributes to some side effects. Non-CPTs—such as indolocarbazoles, phenanthrolines (e.g., ARC-111) and indenoisoquinolines—refer to drugs that have no typical CPT E-ring structures but they can still specifically target TOP1 and bind irreversibly to TOP1cc. Some of the CPT derivatives (*i.e.*, Gimatecan and Belotecan) and non-CPTs (*i.e.*, NSC 725776 and NSC 724998) are presently tested in clinical trials [23].

How does CPT trap TOP1cc? Analysis of the crystal structure and modeling suggest that CPT-TOP1-DNA forms a ternary complex to prevent the two DNA ends from religation [25,26,27]. Although it is still controversial on how CPT is intercalated into DNA, it seems that CPT traps TOP1cc with a thymine (T) at the −1 position and a guanine (G) at the +1 position on the scissile strand, and it is therefore sequence-specific [28]. Three amino acid residues of the TOP1 enzyme, R364, D533 and N722, combined with DNA bases, contribute to the stabilization of the ternary complex by forming hydrogen bonds and hydrophobic interactions. It is of note that several point mutations, including N722S, in *Camptotheca acuminata* TOP1 confer resistance to CPT [29]. Interestingly, the same amino acids also contribute to the inhibition of TOP1 by non-CPT drugs [24].

## 2. Repair of TOP1 Poison-Induced DNA Lesions

As aforementioned, CPT-induced trapping of TOP1cc creates a single strand break with a free 5'-hydroxyl group, whereas the 3'-phosphate is connected to Y723 of TOP1 (3'-P-Y). At least two pathways contribute to the repair of DNA lesions created by TOP1 poison [30]. The tyrosyl-DNA-phosphodiesterase (TDP1) pathway starts with the ubiquitination and proteasome-mediated degradation of TOP1 in the CPT-TOP1-DNA complex to generate a 3'-P end linked to a short peptide [31]. TDP1 then cleaves the P-Y bond to release the 3'-P end; however, the 3'-P end cannot be directly ligated to the 5'-OH end because of the requirements of DNA ligases. The human polynucleotide kinase (PNKP) can process the DNA ends by functioning as both a 3'-phosphatase and a kinase to generate the required 3'-OH and 5'-P termini for direct ligation. The rest of the repair events can be best described by the single-strand break (SSB) repair pathway, which will be discussed below. Indeed, TDP1 and PNKP are tightly associated with the SSB repair machinery [32,33].

The endonuclease pathway requires multiple endonucleases to excise the DNA—usually at a few nucleotides away from the 3'-P-TOP1 end – on the scissile strand to release the DNA-TOP1 complex [30]. Initial studies were carried out to identify genes that functioned in CPT repair in the absence of TDP1 in yeast [34,35]. These studies led to the identification of RAD1-RAD10, SLX1-SLX4, MUS81-MMS4, MRE11-SAE2 as well as genes involved in recombination. The RAD1-RAD10 (human XPF/ERCC4-ERCC1) complex is a DNA structure-specific endonuclease that can act on 5' overhang structures [36]. Interestingly, the cleavage site of XPF-ERCC1 is in the non-protruding DNA strand, about 3–4 nucleotides away from the 3' end [36]. Therefore, trapped TOP1ccs can be removed by this endonuclease activity. Likewise, MUS81-MMS4 (human MUS81-EME1) can also cleave nicked duplex at the 5' of the nick [37]. The SLX1-SLX4 endonuclease, although not tested on nicked duplexes, is able to process 3' flap and other DNA structures [38,39]. In human cells, SLX4 also associates with XPF-ERCC1 and MUS81-EME1 endonucleases to process specific DNA intermediates [39,40]. Moreover, MRE11-RAD50 cleaves the 3'-P-Y bond and resects DNA to produce a 3'-OH end [41]. A direct role of SAE2 (human CtIP) in processing 3'-P-TOP1 is unknown, and its endonuclease activity appears to be limited to the 5' flap or DNA “hairpin” structures [42,43]. Nonetheless, the endonuclease activity of CtIP is essential for processing CPT adducts [42]. In addition, like CtIP, the 5' flap endonuclease RAD27 (human FEN1) seems to be unable to directly process 3'-P-TOP1 ends [44]. However, the gap endonuclease activity of FEN1 is important for processing stalled replication forks and CPT-induced adducts [45]. The role of FEN1 in SSB repair will be discussed further in the next section.

During DNA replication, SSBs created by CPT are most likely converted to double-strand breaks (DSBs) by replication fork runoff. This conversion appears to be dependent on the proteolysis of TOP1 [46]. The repair of one-ended DSBs, as will be discussed in the next section, is largely dependent on homologous recombination (HR). However, low doses of CPT may also induce PARP1 and/or RAD51 dependent replication fork regression—generating no or few DSBs [47,48]. The regressed fork leads to the formation of a “chicken foot” DNA structure by newly synthesized strands [3,49,50]. The formation of regressed fork can be largely suppressed by ATR, EXO1, and DNA2 [51,52,53]. However, fork reversal can also be beneficial as it provides time for the repair of TOP1-induced DNA lesions by TDP1, thereby preventing DSB formation and the activation of error-prone non-homologous end-joining (NHEJ) [30].

## 3. Pathways Involved in the Repair of CPT-Induced DNA Lesions

Normal cells use DNA damage response (DDR) pathways to maintain genomic stability [54]. As aforementioned, SSB and DSB repair mechanisms are the two major DDR pathways that repair TOP1-induced DNA lesions. Paradoxically, cancer cells exploit DDR pathways to accumulate necessary genomic alterations for promoting proliferation. Furthermore, altered DDR and apoptotic responses in cancer cells are the major obstacles to successful chemotherapy. Thus, the delineation of TOP1-related SSB and DSB repair mechanisms is of great importance for identifying drug targets that can selectively affect cancer cell survival.

### 3.1. Single-Strand Break (SSB) Repair

Trapping of TOP1cc results in a 3'-P-TOP1 end and a 5'-OH terminus. Because the two ends cannot be directly religated, the persisting SSB is likely to be detected by PARP1 in which activated PARP1 catalyzes the synthesis of poly(ADP-ribose) (PAR) chains for recruiting repair proteins [55]. This reaction can be rapidly reversed by PARG, which hydrolyzes the PAR chains. The PAR chains at the SSB sites are important for the recruitment of XRCC1 that functions as a loading dock for other SSB repair proteins including TDP1 and PNKP. TDP1 generates 3'-P and PNKP converts 3'-P to 3'-OH, and PNKP also converts 5'-OH to 5'-P, making ends compatible for religation with no base loss. The rejoining of the 3'-OH and 5'-P ends is mainly mediated by LIG3, in which XRCC1 mediates the recruitment of LIG3.

If the trapped TOP1cc intermediates are processed by endonucleases, the initial SSBs will be converted to 3'-OH and 5'-OH ends with a gap over a few nucleotides (in the case of XPF-ERCC1, the loss is in the range of 3–4 nt), leading to the activation of PARP1 and XRCC1 recruitment. Consequentially, Polβ recruited by XRCC1 can catalyze the gap filling, and PCNA-Polδ/ε also plays a role in this process [55]. If the 5'-OH is not processed by PNKP, the 5'-flap resulted from gap filling is likely to be removed by FEN1, which explains why FEN1 deficiency also leads to an increased CPT sensitivity. The final ligation is catalyzed by LIG1 because of the presence of PCNA.

### 3.2. Double-Strand Break (DSB) Repair

Successful DSB repair requires concerted actions of proteins involved in DNA damage signaling and repair [54]. To repair TOP1 poison-induced DNA lesions, ATR signaling is required due to the runoff of replication fork and the presence of long single-strand DNA (ssDNA) [56]. The full activation of ATR follows a “two-man” rule—the ssDNA-ATRIP-dependent recruitment of ATR kinase and the RAD17 clamp loader/9-1-1/TOPBP1 mediator loading at the ssDNA-dsDNA junction. ATR phosphorylates CHEK1 to harness cell cycle arrest. If one-ended DSB is formed, ATM will be activated through the action of the MRE11-RAD50-NBS1 (MRN) complex. ATM mainly phosphorylates CHEK2 to mediate cell cycle arrest. Both ATM and ATR are able to phosphorylate hundreds of proteins in response to DSB formation [57]. One remarkable substrate is the histone H2AX, which can be phosphorylated by both kinases to yield γ-H2AX. It is conceived that the propagation of γ-H2AX signaling along the chromatin facilitates MDC1 recruitment and BRCA1 signaling via the MDC1-RNF8-RNF168-RAP80 ubiquitin cascade—events that are essential for HR [58].

The repair of TOP1 poison-induced DNA lesions is in essence the repair of one-ended DSBs, which facilitates the restoration of replication forks to restart DNA replication. It is important to note that one-ended DSB repair occurs in the S phase and relies on HR rather than NHEJ [59]. The first step in HR is end resection to generate a 3'-overhang for homology searching. A TOP1 cleavage in the leading strand may require end resection by the MRN-CtIP-BRCA1 and BLM-EXO1-DNA2 complexes [60], whereas a cleavage in the lagging strand automatically forms a 3'-overhang. Rad51 then associates with the 3'-ssDNA to form a nucleofilament for strand invasion, which leads to the formation of a D-loop structure [61]. This process continues with DNA synthesis, branch migration and the resolution of Holliday junction structures to reconstitute a functional replication fork [62]. TOP1 poisons can also lead to the formation of two-ended DSB if two replication forks collide into each other at the site of SSB. The repair of this type of DSBs is not aimed for fork restoration and can be accomplished by the classical DSB repair mechanisms [61].

### 3.3. Genes Involved in CPT-Induced Damage Repair

A long list of genes, in which mutations confer sensitivity to CPT in yeast, chicken or mammalian cells, has been compiled [24,30,63]. With no surprise, many genes involved in SSB and DSB repair are on the list, such as PARP1, XRCC1, PNKP, TDP1 for SSB repair; MRN, ATM-CHK2, ATR-CHK1 for DSB signaling; BRCA1/2, XRCC2, XRCC3 for HR. Most recently, the hMSH5-FANCJ complex has also been implicated to play a role in CPT-induced DNA damage response and repair [64]. Mutations in the binding partners of these repair factors are also likely to sensitize cells to CPT treatment. For example, depletion of the MRN-binding partner hnRNPUL increases the sensitivity to CPT [65]; and deficiencies in ZRANB3 and SPIDR, binding partners of PCNA and RAD51, cause CPT hypersensitivity in cancer cells [66,67,68]. In addition, the two DNA helicases BLM and WRN have also been implicated in the repair of CPT-induced DNA lesions [69,70]. Early studies revealed that chicken BLM knockout cells and human BLM-deficient fibroblasts showed increased sensitivity to CPT [71,72]. On the contrary, mouse BLM knockout embryonic stem cells showed mild resistance to CPT [73]. This discrepancy is likely attributable to the complexity of CPT-induced DNA lesion repair as well as different treatment conditions and experimental systems.

Interstrand crosslinks (ICLs) resemble CPT-induced lesions in that they block both replication and transcription [74]. They may induce replication fork reversal and fork collapse, which require DNA incision for lesion processing and HR for repair. ICL repair is accomplished by the coordinated actions of 17 Fanconi anemia (FA) genes whose mutations contribute to FA in patients [75]. Depletion of FANCP/SLX4 or FANCQ/XPF causes cellular sensitivity to CPT because they form an endonuclease complex involved in the repair of trapped TOP1cc [38]. Likewise, depletion of FANCS/BRCA1, FANCD1/BRCA2, FANCN/PALB2 or FANCO/RAD51C sensitizes cells to CPT because of their involvement in HR [76]. Accordingly, depletion of the FA core complex except FANCM—involved in fork reversal—is not expected to increase CPT sensitivity because they are unable to recognize the trapped TOP1cc [76]. However, the roles of FANCI, D2, J and FAN1 in the process are elusive due to conflicting reports presumably reflecting different experimental systems [76,77,78]. For example, in a multicolor competition assay, loss of FANCI or FAN1 rendered cells sensitive to CPT treatment [77]. However, this observation could not be recapitulated in studies performed with FANCI-deficient lymphoblasts and FAN1-depleted HEK293 cells [76,79], indicating that the involvement of these two genes in CTP sensitivity might be cell type specific.

It is interesting to note that the MMS22L-TONSL complex plays a prominent role in mediating CPT sensitivity [80,81,82,83]. Depletion of this complex impairs RAD51 foci formation and triggers G2/M arrest, indicating that the MMS22L-TONSL complex participates in HR repair. Furthermore, this complex associates with MCM, FACT, ASF1 and histones. FACT and ASF1 are histone chaperones that function in H2A/H2B and H3/H4 chromatin assembly and disassembly, respectively [84]. They recycle parental histones from old DNA strands unwound by MCM and incorporate them into newly synthesized DNA strands. FACT and ASF1 also function in checkpoint signaling; therefore the involvement of MMS22L-TONSL in CPT response implies the existence of a close association between HR, DNA damage signaling and replication restart.

## 4. TOP1 Inhibition in Cancer Treatment

The understanding of the function of TOP1 and the cellular effects of TOP1 inhibition has been a stepping-stone for the development of effective CPT derivatives in cancer therapy. Since TOP1 functions in normal and cancer cells, the use of low doses of TOP1 inhibitors are actively sought to treat cancers that heavily rely on the function of TOP1 for survival (e.g., highly malignant, rapid-dividing tumor cells). In fact, the FDA-approved CPT derivatives topotecan and irinotecan are currently used to treat ovarian and colorectal cancers, respectively [24].

Furthermore, the promising results from a Phase I trial have warranted further evaluation of the CPT derivative Diflomotecan in Phase II trials [85]. Other derivatives like Gimatecan, Lurtotecan and Exatecan are also being tested in clinical trials (Table 1). The non-CPT indolocarbazole BMS-250749 showed great anti-tumor activity against preclinical xenograft models [86], but no further evaluation beyond Phase I trials is presently available (Table 2). Another indolocarbazole compound Edotecarin has shown promising anti-tumor activity in xenograft models and it is now advanced to Phase II studies of patients with advanced solid tumors [87]. By contrast, Phenanthroline ARC-111 (topovale) was potently against human tumor xenografts and displayed anti-cancer activity in colon and Wilms’ tumors [88]; however, no result from Phase I clinical trials is available owing to profound bone marrow toxicity [89]. To date, indenoisoquinolines are the most promising non-CPT inhibitors in clinical trials. LMP400 (NSC 743400, indotecan) and LMP776 (NSC 725776, indimitecan) show significant anti-tumor activities in animal models and both are being evaluated in Phase I clinical trials for relapsed solid tumors and lymphomas [8,90].

**Table 1:** CPT derivatives in clinical trials [91].

Name	Structure	Clinical Trial	Malignancy	Reference
Camptothecin	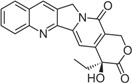	Discontinued		[24]
Topotecan (Hycamtin)	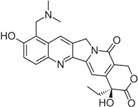	FDA approved	Ovarian cancer, SCLC	[24]
Irinotecan (Camptosar/CPT-11)	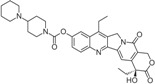	FDA approved	Colorectal	[24]
Belotecan (CKD-602)	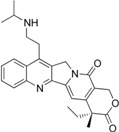	Approved (South Korea)		[4]
Diflomotecan (BN80915)	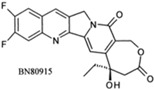	Phase II (Ipsen)	Advanced metastatic cancer, SCLC	[84]
Gimatecan (ST-1481, LBQ707)	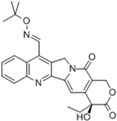	Phase I/II (Sigma-Tau, Novartis)	Advanced solid tumors	[24]
Lurtotecan (Liposomal OSI-211, NX 211)	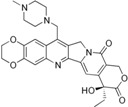	Phase II (Astellas, NCLC)	SCLC, Ovarian	[24]
Exatecan mesylate (DX-8951f)	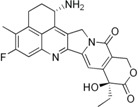	Phase II/III (Daiichi)	Sarcoma, Pancreatic, Gastric, Liver	[24]

Given the observation that CPT-mediated TOP1 inhibition provokes DNA repair activities, a synergistic effect is then anticipated on cancer cells by inhibition of TOP1 and downregulation of DNA repair activities. The rationale for this approach is to accelerate the accumulation of DNA breaks and trigger cellular apoptosis, probably through mitotic catastrophe [92]. Which DNA repair pathways can we exploit? Currently, the major interests are in SSB and DSB repair mechanisms. Indeed, PARP inhibitors can enhance the cytotoxicity of TOP1 inhibitors in cancer cell lines as well as in mouse models [93,94,95,96]. Phase I studies of combination therapy using PARP inhibitors veliparib or olaparib (FDA-approved) together with topotecan were carried out in patients with advanced solid tumors but showed some dose-dependent side effects [97,98]. TDP1 can be another potential target because it functions directly downstream of PARP1 in the repair of TOP1 poison-induced DNA lesions [99]. TDP1 inhibitors sensitize cells to CPT treatment *in vitro* [100,101], however *in vivo* evaluation is presently unavailable due to unsuitable properties of the compounds [102].

**Table 2:** Non-CPT derivatives in preclinical and clinical trials [91].

Name	Structure	Clinical Trial	Malignancy	Reference
Indolocarbazoles (Edotecarin, BMS-250749)	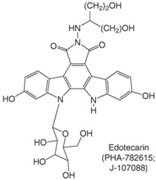	Phase II (Edotecarin, Pfizer) Preclinical (BMS-250749)	Stomach, breast neoplasms Anti-tumor activity in preclinical xenograft models	[86,87,103]
Phenanthridines (ARC-111/topovale)	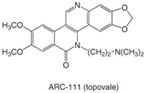	Preclinical	Anti-tumor activity in preclinical xenograft models	[88,89,103]
Indenoisoquinolines (LMP400, LMP776)	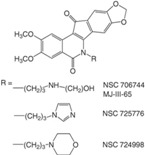	Phase I	Lymphomas	[8,90,103]

DSB repair can be targeted by either inhibition of DSB signaling or inhibition of HR. ATM and ATR inhibitors can largely increase the sensitivity to CPT in cancer cells [104,105]. This can be explained by the fact that abrogation of the cell cycle arrest will allow cells with unreplicated or unrepaired chromosomes to enter mitosis thereby triggering mitotic catastrophe and cell death. Similarly, CHEK1 and CHEK2 inhibitors are tested in Phase I studies in combination with irinotecan [106,107]. Inhibitors that can directly block HR proteins are very limited [108]. This is partially attributed to the fact that HR genes are often mutated in cancer cells, thus diminishing the enthusiasm for developing HR inhibitors. One diterpenoid compound, however, was found to be able to inhibit the function of BRCA1 and render cytotoxicity in human prostate cancer cells [109]. Several RAD51 inhibitors have also been identified but have not been tested in cell lines [110]. Inhibition of BRCA1 and RAD51 can be also achieved indirectly by harnessing corresponding kinases [106]. Clearly, defective hMRE11 sensitizes colon cancer cells to CPT treatment [111]. Although MRE11-deficeint tumor xenografts failed to display significant growth inhibition by irinotecan alone, combining thymidine with irinotecan caused a dramatic growth delay [112].

TOP1 inhibitors might be also useful for treating cancers with BRCA1/2 mutations. The successful use of PARP inhibitors in treating BRCA1/2-deficient tumors has ignited a broad interest in searching for synthetic lethality among DNA damage response and repair genes [113,114]. In the PARP-BRCA1/2 example, the accumulation of SSBs by PARP inhibition would lead to the formation of DSBs during replication. In HR-deficient cells, DSBs can only be repaired by illegitimate (toxic) NHEJ—joining one-ended DSBs from different locations—leading to cell death [115,116]. However, resistance to PARP inhibitors can arise in BRCA1-deficient tumors during treatment from either genetic reversion of BRCA1 mutations or the loss of NHEJ [117,118,119,120,121,122]. Therefore, it would be beneficial to explore the possibility of developing a similar synthetic lethal strategy to use TOP1 inhibitors in the treatment of BRCA1/2-deficient tumors.

**Figure 2:**
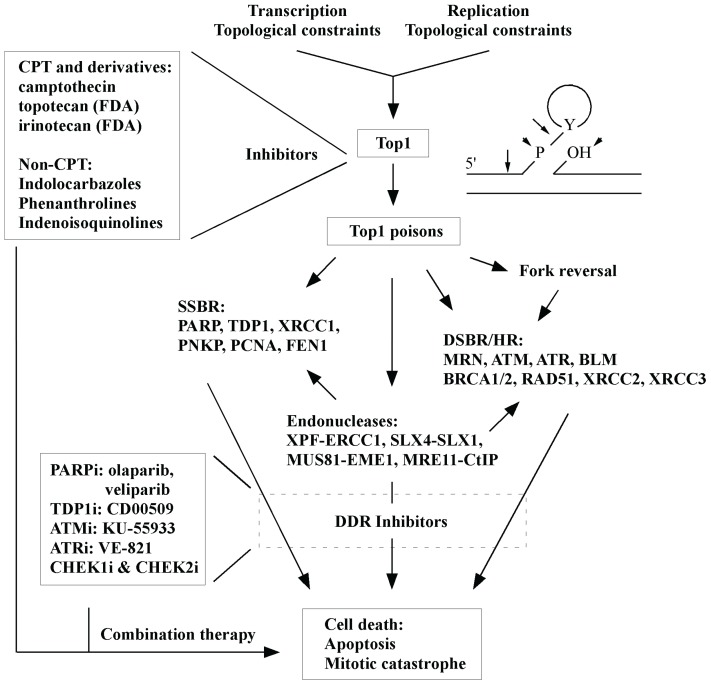
An overview of the effects of TOP1 inhibition is provided. Inhibitors and key DNA repair factors are highlighted.

## 5. Conclusions

Trapping of TOP1 by inhibitors generates SSBs and DSBs that are repaired by their corresponding repair pathways (Figure 2). Therefore, developing effective TOP1 inhibitors not only provides powerful tools to study DNA replication and repair but also establishes a foundation to devise new synthetic lethal strategies for efficient cancer treatments. The accumulation of DNA strand breaks (SSBs and DSBs) by TOP1 inhibition in HR-deficient tumor cells is expected to enhance cytotoxicity. However, increased DNA repair activities in cancer cells can make TOP1 inhibitors less effective, so silencing of repair pathways in conjunction with the use of TOP1 inhibitors offers an attractive new means for cancer control. Since each tumor is unique, it would be advantageous to identify the individualities of DNA repair pathways or biomarkers reflecting the changes of DNA repair activities in tumor cells [92,123]. This will make it possible to achieve better and predictable prognosis through tailored therapeutic regimens. Given that TOP1 is essential for transcription and DNA replication, future design of novel TOP1 inhibitors and combinational therapy strategies should aim to increase therapeutic efficacy of the inhibitors, thus reducing side effects.
